# 

**Published:** 1850-10

**Authors:** 


### CONTENTS.

### LIBRARY.

Page.

A Practical Treatise on Dental Medicine as connected with the Study of Dental Surgery. By Thos. E. Bond, M. D. . 209

### JOURNAL.

Article I. On the Use of Tin as a Base for Artificial Teeth, . 3

Article II. Experiments in the Use of Tin for Insertion of Teeth. By W. A. Royce, Dental Surgeon, of Newburgh, N. Y. . 9

Article III. Filling Teeth after the Pulp-Cavity has become Exposed, . . . . . . . . . . . 12

Article IV. The Touting System amongst Dentists, . . 16

Article V. On the Sensibility of the Teeth. By Alfred A. Blandy, M. D. . . . . . . . . . . 22

Article VI. Importance of Regulating the Teeth, and Employment of Gum Elastic Rings in the Operation, . . 28

Article VII. Adjustment of Clasps, . . . . . . 31

Article VIII. Hints on the subject of Artificial Teeth and Plates, 32

Article IX. Treatment of Exposed Dental Pulp. By H. K. Nutz, Dental Surgeon, Philadelphia, . . . . . 35

Article X. Ninth Annual Meeting of the American Society of Dental Surgeons, held at Saratoga, August, 1848. Reported by William Henry Burr, Phonographer, . . 36

Article XI. Report of the Committee of the American Society of Dental Surgeons, on the Propriety of Rescinding the Amalgam Pledge. . . . . . . . . . 66

### SELECTED ARTICLES.

Page.

ARTICLE XII. Hawes' Moulding Flask, . . . . . . 75
ARTICLE XIII. Regulating Teeth by Spiral Springs, . . 76
ARTICLE XIV. Disease of the Antrum, . . . . . , . . . 79
ARTICLE XV. Case of Disarticulation of the Left Condyle of the Lower Jaw. By W. R. Beaumont, Professor of Surgery, Toronto, Canada, . . . . . . . . 83
ARTICLE XVI. Mr. Gilbert's Fulcrum, . . . 84
ARTICLE XVII. Chemistry and Vegetable Food, . . . 87
ARTICLE XVIII. Partial Deafness of Twenty Years Standing, 89
ARTICLE XIX. New Mode of Reducing a Dislocation of the Lower Jaw, . . . . . . . . 90

### AMERICAN SOCIETY OF DENTAL SURGEONS.

Eleventh Annual Meeting of American Society of Dental Surgeons, 91

### EDITORIAL DEPARTMENT.

New Series of the Journal, . . . . . . 97
American Society of Dental Surgeons, . . . . . 99
Ohio College of Dental Surgery, . . . . . 100
New Drill-Stock, . . . . . . . 101
Gum Elastic in the Treatment of Irregularity of the Teeth, . 102
Premium Teeth, . . . . . . . . 102
A New Preparation of Gold for Filling Teeth, . . . 102
Artificial Teeth set in a Base of Block Tin, . . . 103
Zeiderholz's Self-acting Blow-pipe and Lamp, . . . 103
Correction, . . . . . . . . 104
Nomenclature of the Teeth, . . . . . . 104
Letter from Samuel Rambo, . . . . . . 105
A New File-Carrier, . . . . . . 105
Letter from Warren Rowell, . . . . . . 106
New York Medical Gazette and Journal of Health, . . 106
New York Register of Medicine and Pharmacy, . . 106
Cleaning Plates after Soldering, . . . . . 107
Transylvania School of Dental Surgery, . . . . 107
Filling Teeth after the Lining Membrane has become Exposed, 107
Dental Files, . . . . . . . . 108
Obituaries, . . . . . . . . 108

